# The Agent Preference in Ontogeny: Predictability of Agent and Patient Roles in Child‐Directed Utterances Across Languages

**DOI:** 10.1111/cogs.70147

**Published:** 2026-01-12

**Authors:** Eva Huber, Aylin C. Küntay, Balthasar Bickel, Sabine Stoll

**Affiliations:** ^1^ Department of Interdisciplinary Study of Language Evolution University of Zurich; ^2^ Department of Linguistics University of Cologne; ^3^ Department of Psychology Koç University

**Keywords:** Child language acquisition, Sentence processing, Cross‐linguistic, Neural networks, Word order, Agent preference

## Abstract

Language comprehension unfolds incrementally, requiring listeners to continually predict and revise interpretations. Comprehenders across very diverse languages show a consistent preference for agents, anticipating the agent (“the doer” of an action) more strongly than the patient (“the undergoer”). An unresolved question is how the preference develops in children given incomplete utterances and argument omission in their input. Here, we approach this question by quantifying the incremental predictability of semantic roles (agents vs. patients), probing specifically what kind of contextual information impacts ease of learning. We use transcribed utterances from child‐directed speech in three languages, differing in critical conditions of word order and argument omission: Tagalog (verb‐initial), English (verb‐medial), and Turkish (verb‐final). To quantify incremental predictability at each position in the sentence, we use a computational model trained on naturalistic child‐directed speech, which is first validated against experimental data in each language. Our results show that agents are highly predictable irrespective of sentence position or language, requiring barely any contextual information. In contrast, patient prediction requires additional information, varying by language. These findings suggest that the assignment of agent roles in child‐directed speech is an easier task across typologically distinct languages, possibly reflecting the more general preference for agents outside language. Patients, by contrast, appear to be contextually induced roles that develop in ways that are largely shaped by the affordances of each language.

## 1 Introduction

Human sentence processing is an intrinsically incremental process, whereby comprehenders update their interpretation and representation of a sentence with incoming input (Altmann & Steedman, 1988; Frazier & Rayner, 1982; Tyler & Marslen‐Wilson, 1977). Humans are sensitive to the order in which words are encountered (Bates & MacWhinney, 1989; Ferreira, 2003; Kamide et al., 2003) and the probabilistic relationship between them (Elman et al., 2004; Huettig, 2015; Levy, 2008). This sensitivity is developed early in ontogeny as children gradually acquire parsing strategies tailored to the structural properties of their language, allowing them to interpret language rapidly and accurately in real time (Frazier & de Villiers, 1990; Fodor, 1998; Kidd, 2015; O'Grady, 2005; Phillips & Ehrenhofer, 2015).

Languages vary greatly in how they order constituents in a sentence (Greenberg, 1963; Hawkins, 1983). Consider the event “Anne bakes a cake.” A verb‐medial language like English presents the agent (“doer”) of the action before and the patient (“the undergoer”) after the verb. However, in a verb‐initial language like Tagalog, the action “to bake” is available to the comprehender before the two participants “Anne” and “cake”(“Bakes Anne cake”). In addition, languages vary in word order flexibility and constraints on argument omission (Bickel, 2003; Levshina, 2019; Payne, 1992).

Despite these differences, across languages, both adults and children show a preference to interpret initial role‐ambiguous arguments as agents (e.g., Abbot‐Smith et al., 2017; Bickel et al., 2015; Demiral et al., 2008; Ferreira, 2003; Haupt et al., 2008; Strotseva‐Feinschmidt et al., 2019; Shin, 2021). In 1, the nouns in German are fully ambiguous as to their case and role assignments. In this situation, comprehenders tend to expect “Anna” to be the agent as in 1a and are surprised when “Anna” turns out to be a patient as in 1b.

(1)
  a. …dass Anna          alle            grüßt.
    …that Anna.SG.NOM/ACC/DAT everyone.PL.NOM/ACC/DAT greet.SG
    …“that Anna greets them all.”
  b. …dass Anna          alle            grüßen.
    …that Anna.SG.NOM/ACC/DAT everyone.PL.NOM/ACC/DAT greet.PL
    …“that they all greet Anna.”

These effects are usually measured through event‐related potentials in electrophysiology. They are remarkably robust against differences in word order typology (Sauppe et al., 2023), patterns of case marking (Isasi‐Isasmendi et al., 2024), and persist even after controlling for lexical surprisal (Huber et al., 2024). In line with this, children consistently show higher accuracy scores when interpreting agent‐ rather than patient‐initial sentences (Dittmar et al., 2008; Gertner & Fisher, 2012; Huang et al., 2013; Kolak et al., 2025).

To this date, the agent preference has predominantly been studied in experimental contexts. Experimental stimuli are usually highly controlled in order to avoid confounds and restrict the potential interpretations of the experimental results. As a consequence, they involve structures that are not commonly encountered in children's naturalistic input from which they learn language, such as clauses with two lexical arguments (Haig & Schnell, 2016). Moreover, while most of the world's languages position the agent before the patient in their basic word order (Dryer, 2013), potentially encouraging an agent preference, agents do not consistently appear in the first position in language use. Depending on the grammatical construction and information structure, arguments occur in various positions and word orders differ strongly across languages (Payne, 1992). Although English has a canonical verb‐second word order, patient arguments occur in different positions in child‐directed speech (Cameron‐Faulkner et al., 2003). For instance, the patient follows the verb in imperatives (*eat the banana!*) or may be fronted in certain discourse contexts (*The dog is what I saw*). In other languages like Turkish, agent arguments are often omitted completely when their referent is already given in the discourse (Özge et al., 2019; Underhill, 2003).

Here, we examine how children develop the agent preference as an incremental processing strategy despite this substantial variation in the input to which they are exposed. We select three languages with contrasting basic word orders to investigate how an agent preference might emerge when word order varies maximally. Our sample includes Tagalog, which places the verb first, English, which places it second, and Turkish, which places it last. We simulate the incremental processing and learning of semantic roles using a neural‐network‐based model that we call the “semantic role prediction model.” The model classifies semantic roles and continually updates these interpretations with incoming information. It enables us to quantify at what point, that is, given what kind of information in the sentence, semantic roles can be accurately predicted in naturalistic utterances. The primary objective of the model is thus to quantify position‐dependent semantic role predictability in child‐directed utterances (CDUs) and under the assumption of strictly incremental processing. We note, however, that the model ignores several constraints of online sentence processing (e.g., working memory; Lewis et al., 2006; Boyle et al., 2013) and is, therefore, not intended as a biomimetically realistic model. It only serves as a method to quantify predictability.

The semantic role prediction model proposed here resolves some of the shortcomings of earlier approaches. In particular, semantic role predictability has been extensively studied within the framework of the Competition Model (Bates & MacWhinney, 1989; MacWhinney, 1987, 2018). Such cue‐based approaches generally make predictions about entire sentences or the acquisition of particular features but do not address the incremental, moment‐by‐moment predictions that drive sentence processing (e.g., Chan et al., 2009). In addition, counting the presence or absence of cues in child‐directed speech does not account for the fact that a learner must infer the relevant cues from the input by analyzing the distributional patterns of words and structures in their input. A count‐based approach also requires making choices about which linguistic features to consider, and this limits the comparability of processing across languages that differ vastly in terms of their features. Moreover, comprehenders are highly sensitive to transitional probabilities (Levy, 2008) that are based on their prior linguistic experience, which is not taken into account by a count‐based approach.

The model is trained on transcribed child‐directed speech (child‐directed utterances “CDU”) that are annotated with semantic roles. We first assess whether the model gives realistic predictability measures by qualitatively comparing its predictions to children's behavior reported in experiments. In the main part, we then apply the model to naturalistic CDU in order to quantify role predictability at each position in these utterances. We use Bayesian hierarchical regression models to estimate how the three predictors of sentence position, semantic role (A vs. P), and language affect these predictabilities (technically, the probability that the semantic role prediction model assigns to the correct semantic role). Sentence position is crucial for determining at what point in the sentence roles can be accurately predicted. Comparing roles helps us assess whether predictability differs between A and P arguments. Lastly, including language as a predictor enables us to identify any significant differences in role predictability across the three languages. Apart from allowing a direct test of the weight of these predictors, the use of hierarchical regression models, furthermore, serves to control for the possibility that results based on raw predictabilities (probability measures) are influenced by a subset of sentences.

Throughout this paper, we focus on bivalent (“transitive”) verbs and distinguish between the argument accumulating the most proto‐agent properties (e.g., *volition* or *causing the event*) and the argument accumulating the most proto‐patient properties (e.g., *affectedness* or *undergoing the event*) (Bickel, 2011; Dowty, 1991; Foley & Jr, 1984). We will refer to these roles as A and P subsequently in order to avoid confusion with the more fine‐grained notions of “agent” and “patient” in some grammar theories. In such frameworks, agent and patient usually refer to participants in highly causative events, such as *kill* or *hit* (e.g., Bresnan, 2000). In contrast, our framework uses A and P to refer to the participants of any verb, including verbs like *see*, whose participants would be classified as “experiencer” (here, A) and “stimulus” (here, P) in other approaches. In the remainder of the introduction, we discuss the relevant grammatical features of the three languages and present an outline of the paper.

### 1.1 The languages

#### Tagalog

Tagalog is a symmetrical voice language (Chen & McDonnell, 2019; Riesberg, 2014) in which either A roles or P roles can be made grammatically prominent without any morphosyntactic marking (e.g., the oblique *by*‐phrase in English passives), resulting in the existence of multiple basic transitive constructions. The voice marking on the verb determines whether the syntactically prominent argument, the *ang*‐phrase (glossed here as “SUBJ”), is A or P. For instance, in (2a), the agent voice (“AV”) infix ‐*um*‐ indicates that the *ang*‐phrase is the A role and the *ng*‐phrase (glossed here as “NSUBJ”) is the P of “pulling,” whereas in (2b), the patient voice (“PV”) infix ‐*in*‐ indicates that the *ang*‐phrase is the P role and the *ng*‐phrase is the A role. The voice marking on the verb is thus, in principle, needed to assign roles to arguments.

(2)
  a. H < **um** > i ∼ hila   ng   pusa ang  aso.
< AV > IPFV ∼ pull NSUBJ cat   SUBJ dog.
    “The dog is pulling a cat.”
  b. H < **in** > i ∼ hila   ng   pusa ang  aso.
< PV > IPFV ∼ pull NSUBJ cat   SUBJ dog
    “The/a cat is pulling the dog.”

Word order in Tagalog is relatively flexible. However, there is a tendency for word order in the patient voice to be predominantly A‐initial, whereas the agent voice exhibits more variation (Bondoc & Schafer, 2022; Garcia et al., 2018; Himmelmann, 2005; Hsieh, 2016; Kroeger, 1993; Kaufman, 2009; Schachter & Otanes, 1972). The patient voice is the more frequently used voice both in child‐directed speech (Garcia & Kidd, 2022), as well as in adult‐directed speech (Adricula, 2023).

#### English

English uses word order and to a lesser degree agreement (third person singular ‐*s* as in *she sleep* **s**) and case (only with pronouns) to encode semantic roles. Word order is rigid in that the swapping of two arguments leads to a different interpretation (*The dog chases the cat* vs. *The cat chases the dog*.). It is possible to topicalize the P argument (*the dog I like*) or to front the verb (*Really gets on my nerves, that guy*) for emphasis (Bates et al., 1984), but such word orders are rare in spoken language (Roland et al., 2007). Similarly, the passive voice, in which P is placed first, occurs infrequently in spoken language and predominantly appears without the A expressed as an oblique phrase (Xiao et al., 2006). In child‐directed speech, imperatives and *wh*‐questions are frequent (Cameron‐Faulkner et al., 2003), which introduce word orders other than AVP into the linguistic input to children.

#### Turkish

Consistent with typological trends, Turkish, as a verb‐final language, relies heavily on case marking to encode roles. It makes use of both verbal as well as nominal indexing to encode semantic roles. The A role is usually unmarked (nominative case) and P is often marked with the accusative case (Example 3a) (Slobin & Zimmer, 1986). However, case marking for the accusative case is optional for generic and, depending on the context, indefinite objects (Example 3b) (Underhill, 2003). The encoding of A arguments involves additional complexities, as they can also appear in embedded clauses in the genitive case (Göksel & Kerslake, 2004; Özge et al., 2015).

(3)
  a. Bu **parça‐yı**  ancak  çok  iyi  bir **piyanist** çal‐abil‐ir.
    this piece‐ACC only  very good a  pianist  play‐PSB‐AOR
    “Only a very good pianist can play this piece.” (Göksel & Kerslake, 2004, p. 128, 14)
  b. **Bir** mektup yaz‐dı‐m.
    a  **letter**   write‐PFV‐1SG
    “I wrote a letter.”(Göksel & Kerslake, 2004, p. 128, 14)

While the basic word order is APV, word order in Turkish is flexible and determined by information structure (Erguvanlí & Taylan, 1984). Argument omission is common in Turkish, and any argument inferrable from the context can be omitted. Omission of the A argument is especially prevalent, which makes P‐initial orders frequent and discourse‐neutral (Demiral et al., 2008; Özge et al., 2019).

The grammatical features of the three languages suggest different position‐dependent role predictabilities. In Tagalog and Turkish, word order is not a necessary cue for semantic roles, since verbal and nominal marking disambiguate semantic roles irrespective of their linear order. The voice marking on Tagalog verbs (when present) unambiguously identifies the roles of the arguments. In Turkish, case marking on nouns (when present) is a relevant cue for semantic roles, disambiguating the roles of the arguments before the arrival of the verb in verb‐final sentences. In English, however, marking on the argument is not available apart from pronouns, and comprehenders have to focus on word order and/or agreement. Thus, in Tagalog and Turkish, predictability of A may be less dependent on the argument's position. In contrast, in English, predictability of A is more closely tied to its initial position. While A‐before‐P word orders are predominant in naturalistic speech in both Tagalog (Adricula, 2023; Garcia and Kidd, 2022) and English (Xiao et al., 2006), Turkish often omits A (Demiral et al., 2008). Thus, in Turkish, a parser may not be able to rely on the first argument to be A. Despite this, adults show an agent preference and transiently misinterpret initial P arguments as A arguments when processing role‐ambiguous (case‐less) arguments (Demiral et al., 2008); however, it remains open whether this bias extends to naturalistic input to children. Another open question is whether pre‐verbal A arguments can be equally well predicted across languages. In English, this is the default word order. However, in Tagalog, voice morphology on verbs disambiguates the role of an argument, and, as a consequence, the parser may only be able to accurately assign the role once the verb is encountered.

### 1.2 Outline

We first describe the semantic role prediction model, the datasets, annotation, and statistical model in Sections 2.1, 2.1.1, 2.1.2, 2.1.3, 2.1.4, 2.2, [Link], Model architecture, Model training, 2.3. Section 3 presents the results: first, we evaluate whether the model's role predictability estimates are realistic and sensible by comparing them qualitatively to children's behavioral results reported in experiments.

Next, we apply the model to CDUs and extract the predictabilities in the form of correct role assignment probabilities at different time points in the sentence. Finally, we regress these predictabilities on sentence position, role (A vs. P), and language, allowing for variation across sentences in a Bayesian hierarchical model. The presentation of the results will be followed by a discussion (Section 4) and a conclusion (Section 5). The scripts and Supporting Information are available under https://osf.io/e8jk4/?view_only=278b83bd88e040c397a25d25cf7b023f.

## 2 Methods

### 2.1 Data and annotation

Our main datasets of CDUs are compiled from child language corpora of the three languages. We use additional datasets taken from corpora of adult conversations for pretraining the semantic role predictability model (see description of the model below in Section 2.2 (adult‐directed utterances [“ADUs”])).

#### 2.1.1 Child language corpora for the CDU dataset

For the CDU datasets, we extract utterances with bivalent (“transitive”) verbs, that is, verbs with two arguments, from transcriptions of child speech and their surrounding speech. For Tagalog, we use the corpus by Garcia and Kidd (2022), which contains 1‐h recordings of interactions between 20 children, aged 2;0–4;0 years, and their caregivers in their homes. The CDU data for English is taken from the MPI‐EVA‐Manchester Corpus (Lieven et al., 2009) and the Manchester Corpus (Theakston et al., 2001), two longitudinal corpora that track the child language development of 16 children aged 1;8–5;1 years. For Turkish, we use a longitudinal corpus of Turkish (Jancso et al., 2020; Küntay et al., unpublished) that includes interactions between eight children and their caregivers. Children's ages range from 0;8 to 2;9 years.

**Fig. 1:**
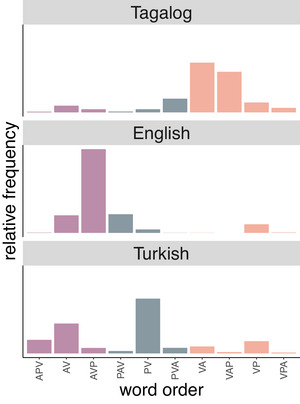
The relative frequencies of word orders in the child‐directed utterance (CDU) datasets for each language with A=Agent, P=Patient, and V=Verb grouped and color‐coded by the sentence‐initial element.

The distributions of word orders in the datasets used in the present study exhibit the expected variability (Fig. 1). In the Tagalog dataset, A‐initial utterances are more frequent than P‐initial (75.8% of utterances). While verb‐initial word orders are most common, word orders with preverbal arguments also occur. For instance, P‐initial word order is common in questions (*ano* (what.P) *isusulat* (“are going to write”) *mo* (“you.A”) *diyan* (“there”)? (Garcia and Kidd, 2022, 285)), or topicalizations of P (*bird* (bird.P) *tingnan* (look) *mo* (you A) *oh!* (Garcia and Kidd, 2022, 1126)).

In the English dataset, A‐initial word orders are prevalent (76.5% of the utterances in our dataset), but P‐initial utterances also occur (21.9%), particularly with questions (“What are you doing?” (Lieven et al., 2009, 419412 in)) or imperatives where the A argument is usually not expressed (“look at this!” (Lieven et al., 2009, 418771)).

In Turkish, the most frequent word order is “PV” showing that the A argument is often omitted. As a consequence, the number of A‐initial sentences is considerably lower than in Tagalog and English (43.0%). However, when the A is expressed in Turkish, it does often occur in first position (86.6% of sentences with A arguments). Verbs regularly appear in initial, medial, and final position.

As expected, word order varies considerably less in English than in the other two languages, shown by the entropy of word order of each language based on our dataset (Tagalog: 1.66, English: 1.15, Turkish: 1.72).

#### 2.1.2 Annotation

We only analyze clauses with “bivalent” (or “transitive”) verbs. We define verbs as bivalent following a semantic definition of valency. The verb's valency is not defined based on the syntactic status of its arguments and adjuncts (i.e., whether they are a subject, object, or an oblique) but on the argument's semantic relation to the verb. Instead, the valency is determined by the number of arguments it semantically entails (Dowty, 1991). For instance, the verb *eat* is considered bivalent because the verb entails the presence of someone eating and something being eaten, regardless of the fact that it can be used both transitively and intransitively (with a generic patient). Similarly, *go* is considered bivalent since it entails that someone or something moves to a certain goal location (as can be seen in the interpretation of “where” as a goal and not as a static location in “where do you go?”, compared to “where do you dance?”). Under a syntactic definition of valency, *go* would be a monovalent verb as the goal argument is mostly expressed with the preposition *to*. Defining the valency of verbs based on their semantic entailments is favorable when comparing languages since the expression of arguments of verbs either as objects or obliques hugely varies across verbs. For instance, the P argument “her aunt” of “to visit” is expressed as an object in English (“She visits (*to) her aunt”), but as an oblique (*sa*‐phrase and not *ng*‐phrase) in Tagalog (“Dumadalaw (< AV > IPFV ∼ visit) siya (3SG.SPEC) **sa**/*ng kanyang (her) tita (aunt)”).

The arguments of the verbs were then annotated with “proto‐roles” (Dowty, 1991). The argument of a bivalent verb that accumulates more agent properties (e.g., “causing an event,” “volitional,” “sentient”) is considered the A role of an event. The argument accumulating more P properties (e.g., “undergoing an event,” “not independently existing”) is considered the P role of an event. For instance, in “Mary baked a cake,” “Mary” the A argument is volitional and causes the baking event, whereas “the cake,” the P argument, does not independently exist and undergoes a change of state.

For English, we used the semantic role parser of AllenNLP (Gardner et al., 2018) to annotate the arguments with semantic roles. This semantic role parser is trained on data annotated by the PropBank annotation scheme that follows Dowty (1991)'s proto‐role method. The utterances were then checked by two proficient speakers of English. We selected only the verbs that both annotators agreed to be bivalent. The Turkish utterances were manually annotated by Turkish native speakers. For the Tagalog dataset, we adopted the annotations provided by Garcia and Kidd (2022). In order for the annotations to be consistent with our definitions of valency and roles, we made minor changes in consultation with native speakers. For instance, we changed the valency of verbs that often omit the P argument, such as *kain* “to eat,” from monovalent (“intransitive”) to bivalent (“transitive”). The datasets contain the following number of clauses with a verb and at least one argument: 5382 for Tagalog, 3065 for English, and 4847 for Turkish.

#### 2.1.3 Experimental stimuli

We evaluate to what extent our role predictability estimates are realistic by applying the model to stimuli of previously conducted experiments that test children's knowledge of cues for semantic roles. If our model estimates are realistic, we expect them to mirror children's performance in assigning A and P roles in these experimental stimuli. See Table 1 for an overview of the experiments and results. All of the studies are based on bivalent verbs and apply the same binary categorization of roles into A and P as in our model. However, the stimuli only include verbs with prototypical As or Ps, such as “pull,” where the A triggers some physical reaction in the P. Thus, unlike the data that the model is trained on, the experimental stimuli only include a fraction of bivalent verbs. With the exception of English, the experimental designs for Tagalog and Turkish do not involve case‐ambiguous initial patients. Consequently, we do not expect a consistent agent preference in these experiments, as children have access to cues indicating the patient.

**Table 1:** Overview of experimental conditions and results of behavioral tasks for Tagalog and English, and online eye‐tracking results for Turkish

Language	Age groups	Condition	Result
Tagalog (Garcia et al., 2020, 2021)	5‐ and 7‐year‐olds	Agent Voice, A‐initial	High accuracy in both age groups
		Agent Voice, P‐initial	Low accuracy in both age groups
		Patient Voice, A‐initial	High accuracy in both age groups
		Patient Voice, P‐initial	Above‐chance accuracy in both age groups, higher in older age group
English (Abbot‐Smith et al., 2017)	25‐ and 41‐month‐olds	Active (A‐initial)	Above‐chance accuracy, higher in older age group
		Passive (P‐initial)	Low accuracy in younger group; above‐chance, modest accuracy in older group
Turkish (Özge et al., 2019)	4‐year‐olds	Verb‐medial, nominative case (A‐initial)	Correct prediction of second argument after seeing the verb
		Verb‐medial, accusative case (P‐initial)	Correct prediction after verb; bias toward interpreting initial argument as agent
		Verb‐final, nominative case (A‐initial)	Correct prediction of second argument before verb appears
		Verb‐final, accusative case (P‐initial)	Correct prediction of second argument before verb appears

*Note*. (Tagalog: Garcia et al. (2020, 2021); English: Abbot‐Smith et al. (2017); Turkish: Özge et al. (2019)). A refers to the agent role and P refers to the patient role.

For Tagalog, we compare our model's predictabilities to the study by Garcia et al. (2020) that tested 5‐ and 7‐year‐olds' comprehension of roles with bivalent verbs, manipulating voice (agent voice vs. patient voice), and word order (A‐initial vs. P‐initial). Table A1 in the Appendix contains examples for each condition. Participants completed a picture selection task featuring two reversible actions while their looks to the pictures were tracked. The authors found that children of both age groups comprehended A‐initial sentences more easily than P‐initial sentences in both voices. P‐initial sentences in the agent voice were most difficult to understand, again for both age groups. Note that the children are older than the ones recorded in the corpus we use, limiting the agreement we can expect between studies. We use the stimuli from a later paper by the same first author (Garcia et al., 2021) because the stimuli in Garcia et al. (2020) are not publicly available. The stimuli only vary in certain items to meet the needs of the paradigm and do not differ in complexity, so this should not impact the comparability.

The English stimuli come from Abbot‐Smith et al. (2017), who examined children's processing of active (A‐initial) and passive sentences (P‐initial) to determine whether they exhibit the tendency to interpret initial noun phrases as agents and if they can override this tendency. Examples for each condition are provided in Table A2 in the Appendix. They conducted a preferential‐looking experiment with eye‐tracking as well as a forced‐choice pointing experiment with 2‐ and 3‐year‐olds. Both age groups exhibited a bias to interpret the first noun phrase as the A argument. Only the older children reanalyzed their initial misinterpretation of the first argument being the A in the passive voice and, to some extent, distinguished the active and passive voice in the forced‐choice pointing task. The experiment was conducted with children within the same age range as the children featured in the English corpora.

Lastly, for Turkish, we use the stimuli by Özge et al. (2019). In this study, the authors tested whether 4‐year‐old Turkish‐speaking children can use case marking on the first noun to predict the upcoming referent of the second noun. They used the visual world paradigm and tracked children's gazes toward possible referents in two experiments. In both experiments, the case marking of the first noun, that is, its role, was manipulated (A [nominative] vs. P [accusative]). In the first experiment, the word order was verb‐medial (AVP vs. PVA), whereas in the second experiment, the word order was verb‐final (APV vs. PAV). Examples for each condition are again provided in the Supplementary Appendix, in Table A3. They found that 4‐year old children predict the upcoming referent before the arrival of the second noun on the basis of case‐marking on the initial noun. In the verb‐medial word order, children only did so after seeing the verb. Children also exhibited a bias toward interpreting the first NP as the A argument. The study was conducted with older children than the ones featured in the Turkish corpus we use to train the model. This, as for Tagalog, lowers the expected degree of agreement between the experimental results and the model predictions.

#### 2.1.4 Adult conversations for pretraining (ADU)

We first pretrain parts of the semantic role prediction model on spoken language corpora of adult interactions, called adult‐directed utterances (“ADU”) hereafter. The pretraining stage is conducted to ensure that the model acquires a basic knowledge of words and their transitional probabilities. We use adult spoken corpora rather than ecologically more valid corpora of child‐directed speech because of the severe size limits in transcribed child‐directed speech. In addition, child‐directed speech mostly contains short utterances (e.g., mean of 2.6 in the Tagalog corpus) and many of the utterances do not contain verbs (e.g., only 23.22% of utterances in the Tagalog corpus contain verbs), likely restricting the linguistic knowledge and lexical diversity in the model's input. Since any corpus of child‐directed speech only captures a small fraction of a child's linguistic experience, we do not think the addition of adult interactions to the model distorts the results. Furthermore, the focus of this paper is comparative. Since we apply the model on the same types of data across languages, we ensure that the results remain comparable across languages. For English, we use the spoken part of the British National Corpus (BNC) (Love et al., 2017), which consists of transcribed spontaneous conversations among native speakers of British English from across the UK. For Tagalog and Turkish, we use the IARPA Babel Tagalog/Turkish Language Pack (Andresen et al., 2016; Bishop et al., 2016). These datasets contain ∼200 h of transcribed telephone conversations between adult native speakers of each respective language in different settings. From each of these corpora, we select a random sample of utterances comprising 200K words that forms our training set to pretrain the semantic role model on the next word prediction task (number of utterances for Tagalog: 16,796, English: 18,742, and Turkish: 21,368). We also experimented with larger training sets but found that this did not improve the final results for semantic role classification. However, we note that this number is significantly lower than what a child is likely to be exposed to (Frank, 2023; Gilkerson et al., 2017; Hart & Risley, 1992).

### 2.2 The semantic role prediction model

We implement a semantic role prediction model designed with specific architectural components to fulfill key requirements essential to address the main research questions of this paper. The primary objective of the model is to determine the probability of each role at a particular point in the sentence based on the already encountered input, assuming incremental processing but without imposing additional plausibility constraints. In other words, similar to human comprehenders, the model should progressively construct a representation of “who does what to whom” as each word is encountered. An important aspect thereby is that the model should be able to update earlier predictions since humans constantly predict upcoming input based on already processed input during language processing (Kuperberg et al., 2020; Schrimpf et al., 2021). An additional product of incrementality should be sensitivity to transitional probabilities between words, enabling the parser to anticipate likely upcoming elements. Furthermore, in order to study how parsing strategies develop based on naturalistic input, the model should learn from naturalistic input and not rely on in‐built features, for example, information on selectional restrictions of verbs. In addition, we want a graded measure in the form of the probability of the correct semantic role, rather than a binary value of correct or incorrect, allowing us to assess not only whether the semantic role can be identified, but also the degree of certainty with which a parser can make that interpretation. Lastly, the model should be language‐agnostic in the sense that it should not be tailored to a particular language and should be able to explain linguistic patterns across diverse languages. As such, our model is a model of grammar and language use on the sentence level, and it does not include information on discourse‐pragmatics, prosody, and extralinguistic cues, such as visual information.

Several models have been proposed in the past to explain language acquisition and processing in relation to semantic roles (see for an overview McCauley & Christiansen, 2014). These models have provided insights into possible mechanisms of semantic role learning. However, they often lack cross‐linguistic applicability (Alishahi & Stevenson, 2010; Chang et al., 2006) or do not process language incrementally (Connor et al., 2013; Huebner et al., 2021), both of which are essential prerequisites for our desired model. Our model is inspired by the model of Hörberg and Jaeger (2021), which targets word‐level updating in semantic role interpretation. However, it does not sufficiently meet our criteria because it predicts the structure of the whole sentence (i.e., AVP vs. PVA) instead of individual roles. Additionally, the Sentence‐Gestalt model by Lopopolo and Rabovsky (2024) provides a connectionist model that makes predictions over the sentence meaning in the form of argument structure. However, the classification of each role happens only implicitly, whereas we need to directly access the probabilities of each role at each time point.

**Fig. 2:**
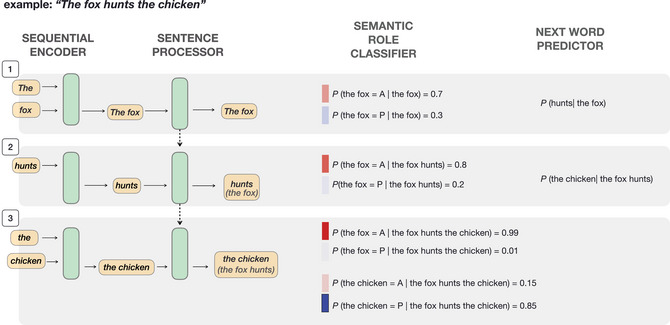
An illustration of how roles are classified within the semantic role prediction model based on the toy example “The fox hunts the chicken.” The model consists of a *sequential encoder*, a recurrent neural network (RNN), that reads in the vector representations of strings (e.g., “the” and “chicken”), and creates one representation for arguments that consist of multiple words (e.g., “the chicken”) and a *sentence processor*, also an RNN, that processes each word and argument and creates a representation of the sentence up to the current time point (e.g., “the chicken (the fox hunts)”). This representation is then fed to the *semantic role classifier*, which classifies the roles of already seen arguments given the words up to the current time point. The representation is further fed to the *next word predictor*, another classifier which predicts the upcoming word.

In order to meet the criteria specified above, our semantic role prediction model consists of three main parts (see Fig. 2): (1) a *sentence processor* that encodes words one at a time; (2) a *semantic role classifier* that predicts the semantic role of the current and preceding arguments based on the current input; and (3) a *next word predictor* that predicts the next word given the already encountered words. The sentence processor is necessary to model the incremental nature of sentence processing and the engagement in predicting upcoming input. The semantic role classifier is necessary for estimating the probability of a role based on the current position as well as allowing the model to revise the role predictions in earlier arguments based on the current input. Finally, the next‐word predictor is essential for capturing the linear probabilities between words, a task which is not covered by the semantic role classifier, but necessary for the model to learn the common sequences of the language. Here, we provide a conceptual explanation of the model. For a more detailed explanation, please refer to the detailed model description included in the Appendix (Section B).

##### Model architecture

**Fig. 3:**
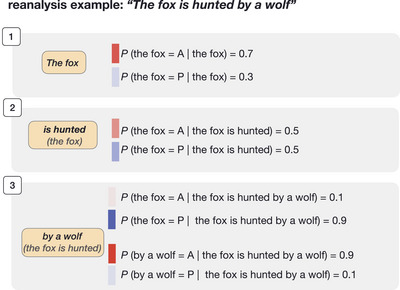
A toy example illustrating how the semantic role prediction model enables reanalysis of earlier interpretations. Initially, the model assigns a higher probability for A for “The fox.” Upon encountering “is hunted,” this probability decreases and finally reverses to a higher probability for P once the actual A argument, “by a wolf,” is encountered.

The words of a particular utterance (e.g., “the fox hunts the chicken” in Fig. 2) are first transformed into vector representations. We used two types of vector representations. First, we tested a Convolutional Neural Network (CNN) layer that calculates a representation of a word based on its characters and, second, we tested pretrained fastText embeddings (Bojanowski et al., 2017). The vector representations of individual tokens are then fed to the sequential encoder. For arguments with multiple words (e.g., “The fox”), the sequential encoder creates a representation of the entire argument. In this way, we ensure that the semantic role is later predicted based on the entire argument, and not just on its head (e.g., *fox*). Words that are not part of arguments (e.g., “hunts”) are represented alone. The sentence processor, implemented as a recurrent neural network, specifically a long short‐term memory (Hochreiter & Schmidhuber, 1997), creates a new representation of the word(s) as a function of the current word (e.g., “hunts” at time point 2) and the already encountered words (e.g., “The fox” at time point 1), allowing the model to be sensitive not only to the current word, but also to incrementally build a representation of the entire sentence. The output of the sentence processor (e.g., “the chicken (the fox hunts)” at time point 2) is then directed to two classifiers. The first classifier, the next word predictor, is trained to predict the next word (e.g., the word at time point 3 based on “the fox hunts”) (Fig. 2). This task is generally known as language modeling, which helps the model to become sensitive to probabilities between words, and as a result, acquire general linguistic knowledge (Linzen & Baroni, 2021; Jurafsky & Martin, 2025). Second, the output from the sentence processor is also fed to the semantic role classifier. The semantic role classifier classifies the semantic role of an argument based on a representation of the argument to be classified (e.g., at time point 2: “the fox”), a representation of the current word (“hunts (the fox)”), and a representation of the verb if it has already been encountered in the input (“hunts (the fox)”). At each time point, the classifier predicts the roles of all of the arguments that have already been encountered in the input, for example, in the example in Fig. 2, the semantic role of “the fox” is classified at each time point as it has already been observed at time point 1. As a result, earlier classifications can be reanalyzed (see “reanalysis example” in Fig. 3). Additionally, the roles of both arguments are classified independently, such that, for example, the classification of “the fox” at time point 3 is not conditioned on the predicted role of “the chicken.” For sentences containing a single argument, only the semantic role of that argument is predicted. Both classifiers are implemented as one‐hidden layer neural networks.

##### Model training

The model is trained both on the next word prediction task as well as the semantic role classification task. The error between the predicted and target semantic roles is computed at the final sentence position, while the error between the predicted and actual next word is calculated at each position throughout the sentence. The training is conducted in two stages. In the pretraining stage, the model is only trained based on the next word prediction classifier, that is, without the semantic role classifier. We conduct this pretraining stage to allow the sentence processor to become sensitive to probabilities between words and acquire a basic knowledge of the language. For this pretraining stage, the corpora of spoken languages are used (see Section 2.1.4). In the main training stage, we train the whole model, that is, the semantic role classifier in Fig. 2 is trained in addition to the sentence processor and next word predictor. This main training stage is conducted on the utterances extracted from the CDU dataset.

We also experimented with training only the semantic role classifier in the main training stage without the next word prediction task. However, training both the semantic role classifier and the next word predictor together resulted in higher role classification scores. We evaluate the sentences using 10‐fold cross‐validation. This involves splitting the dataset into 10 different configurations of training, validation, and test sets, ensuring that each utterance in our dataset appears once in the test set. This allows us to analyze our entire datasets and not only a subset of the data.

We trained the semantic role prediction model on both types of vector representations (fastText and CNN). With both the experimental stimuli and our dataset, fastText embeddings slightly outperformed the CNN embeddings in accuracy (see accuracies in 1.1 and 2.2 Supporting Information). Therefore, we will only report the results based on the fastText embeddings.

### 2.3 Regression model

We then analyze how the semantic role predictability varies by sentence position, semantic role, and language using regression models. In other words, we treat the output of the semantic role prediction model analogous to, for example, the responses of a human participant in an experiment.

For both the analysis of the experimental stimuli as well as the analysis of the naturalistic utterances from child‐directed speech, we fit hierarchical Bayesian beta models to estimate the probability of the correct semantic role assigned by the semantic role prediction model in *brms* (Bürkner, 2017, 2018, 2021). See the Appendix C for a mathematical model notation. For the analysis on the experimental stimuli, we only include the probability that the model assigns to the correct role in the last position in the sentence, meaning the final prediction of the model, and regressed this on role (A vs. P) and experimental condition(s) (Tagalog: voice [agent voice vs. patient voice] and word order [A‐initial vs. P‐initial]; English: voice [active vs. passive], Turkish: role of first noun phrase [A vs. P] and word order [AVP vs. APV]). We further add interactions between role and the condition(s) as well as random intercepts slopes for condition(s) and role per stimuli ID.

For the analysis of the CDU dataset, we include the predictions at up to three different sentence positions. For instance, the probability of the role A in a sentence with word order AVP is included three times: at its first occurrence, at the occurrence of the verb, and at the occurrence of P. On the other hand, the probability of the role P is only included once since it is only accessible to the semantic role processing model at the end and, therefore, there is only one probability. In the regression model, we fit sentence position, role, and language as main predictors of interest. We code position with two binary values that indicate whether, first, the argument to be predicted occurs after the other argument (*after argument*) and second, the argument occurs after the verb (*after verb*), with *after argument* always being False in case there is only one argument. We further add an interaction between *role*, *after argument*, and *after verb*, which enables us to model all possible word orders. Additionally, the form of the argument and the type of verb likely influence role predictability, thus, we also add predictors of *nominality* (pronoun of 1SG, pronoun of 2SG, other pronouns, other [mostly lexical noun phrases]) and verb classes. The verb class consists of an inventory of 48 verb classes taken from AUTOTYP (Bickel et al., 2022). Examples are *perception*, *motion*, and *existence*. We included multiple interaction terms between the main effects to account for potential correlations among them. For instance, a correlation between the form of the argument (*nominality*) and *role* is expected, as As are more frequently expressed as first or second person pronouns compared to Ps (Silverstein, 1981). In our datasets of child‐directed speech, 67.4% of A arguments and 5.7% of P arguments are first or second person pronouns in the Tagalog dataset, 47.1% of A arguments and 8.0% of P arguments in the English dataset, and 30.3% of A and 5.8% of P arguments in the Turkish dataset. By including a four‐way interaction between *after argument*, *after verb*, *role*, and *language*, we account for the possibility that in some languages one type of combination of position and role may be more prevalent than in another language. We included random intercepts and slopes for *after argument*, *after verb*, and *role* per sentence ID. For the model on the CDU datasets, we also tried to fit a model including a random slope for the interaction between the three terms; however, it did not converge. We use weakly informative Normal(μ = 0, σ = 1) priors for the population‐level slopes and Half‐Normal(μ = 0, σ = 10) or Exponential(λ = 1) priors for the precision parameter ϕ (Half‐Normal(μ = 0, σ = 10), or Exponential(λ = 1), depending on which leads to better model fit). For the by‐sentence variation of the intercept and slopes, we use the default priors of brms, a Student‐*t*(df = 3, μ = 0, σ2.5) distribution. The model fit is evaluated based on the $\hat{R}$ ($\hat{R}$ <1.01) and effective sample size diagnostics (100 times numbers of chains, that is, ESS >400, as recommended by Gelman et al. (2013) and Stan Development Team (2021)).

To ensure that the particular language and the position of the argument within the sentence are relevant for semantic role prediction, we perform model comparison between the full model and three more models that lack one of the main predictors: one without *after argument*, one without *after verb*, and one without *language*. We conduct leave‐one‐out cross validation to calculate the expected log pointwise predictive density (*elpd*) and then apply model stacking to compare the individual performance of the models (Yao et al., 2018). We find that the full model is assigned the highest weight (98%), indicating that all of the predictors are relevant to accurately estimate role predictability (see Appendix D for a more detailed description of the approach and results).

## 3 Results

Here, we present the results from the fitted regression models on role predictability as estimated by our semantic role prediction model in terms probability of correct role assignment. We first discuss the results of the experimental stimuli (Section 3.1), and show that the semantic role prediction model returns predictabilities that are in line with children's sentence processing. Having assessed the reliability of the semantic role prediction model, we then present the results of the CDU datasets (Section 3.2).

### 3.1 Role predictability in experimental stimuli

**Fig. 4:**
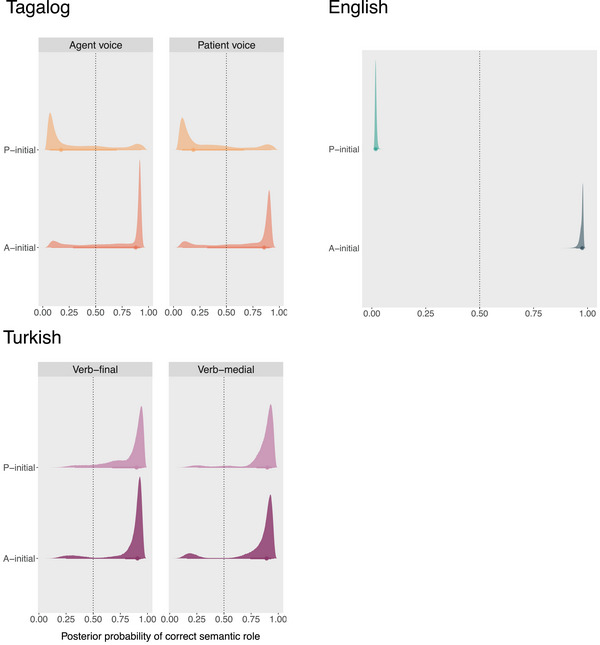
Results of the models evaluated on the experimental stimuli. For each language, the plots show the posterior distributions (mean, 66% and 95% credible intervals) of the semantic role prediction model's assigned probability to the correct semantic role at the last position in the sentence, averaging across roles. In Tagalog, the mean is above chance for the A‐initial stimuli, but the credible intervals are large, showing the low confidence with which A‐initial sentences are predicted. P‐initial stimuli are not predicted accurately. In English, the mean in the active voice is at ceiling, but below average in the passive voice. For Turkish, means are above chance in all conditions, but the 95% CI includes areas below 50% indicating that the predictability strongly varies across stimuli.

Fig. 4 displays the posterior probabilities of the correct semantic roles in the stimuli from the experiments for each language.

For Tagalog, our model‐based role predictabilities are consistent with experimental findings in all conditions except for the P‐initial, patient voice conditions. Mean predictability (i.e., the mean probability of classifying the correct role) in the A‐initial conditions are above chance in both the agent and the patient voice; however, the 95% credible intervals (CI) are large (posterior mean P(correct role|A-initial, agent voice)=0.69,95%CI=[0.08,0.94]; posterior mean P(correct role|A-initial, patient voice)=0.69,95%CI=[0.09,0.93]). This is in line with the results of the picture selection task in Garcia et al. (2020), where children showed high performance with A‐initial sentences. In the P‐initial conditions, predictions are below chance, but the credible intervals cover almost the whole range of possible values (posterior mean P(correct role|P-initial, agent voice)=0.32,95%CI=[0.04,0.93]; posterior mean P(correct role|P-initial, patient voice)=0.32,95%CI=[0.05,0.92]). Similarly, 5‐year‐old children (the youngest group) scored below chance for P‐initial sentences in the agent voice. Unlike our model, they performed above chance with stimuli in the P‐initial patient voice condition but lower than with both A‐initial conditions. This trend persists among 7‐year‐olds. The model was trained on utterances directed to younger children than the children tested in the experiments. This mismatch in age likely accounts for the discrepancies in performance between the semantic role model predictions and children in the P‐initial, patient voice. Children tested in the experiments have already acquired the voice marker on the verb in the more frequently occurring patient voice, and thus, do not misinterpret first‐occurring Ps as As.

Regarding the English experiment, the semantic role prediction model exhibits a strong agent preference that leads to a misinterpretation of P‐initial sentences in the passive voice. A similar pattern emerged among the children in the experiment (Abbot‐Smith et al., 2017). The posterior probabilities of the correct semantic roles in the active voice (AVP word order) are at ceiling (posterior mean P(correct role|active voice)=0.97,95%CI=[0.95,0.98]), while in the passive voice, they are at floor level (posterior mean P(correct role|passive voice)=0.02,95%CI=[0.01,0.03]), indicating that the model processed passive voice like active voice. In Abbot‐Smith et al. (2017), 2‐year‐old children performed around chance, while 3‐year‐old children performed slightly above chance in both conditions; however, both age groups show an incremental bias toward interpreting the first noun as A, in line with our model predictions.

The modeling results for Turkish are in line with the experimental results reported in Özge et al. (2019). The role predictabilities in the Turkish experimental stimuli are above chance in all conditions, but, as for Tagalog, the credible intervals are rather large (posterior mean P(correct role|verb-final, A)=0.84,95%CI=[0.24,0.96]; posterior mean P(correct role|verb-final, P)=0.82,95%CI=[0.33,0.97]; posterior mean P(correct role|verb-medial, A)=0.81,95%CI=[0.16,0.96]; posterior mean P(correct role|verb-medial, P)=0.84,95%CI=[0.26,0.97]). Consistent with our model‐based predictabilities, children show good command of using case marking for role disambiguation as they can predict the referent of the second argument based on the case marking on the first noun. The uncertainty in the modeling results, shown by the relatively large credible intervals, may again stem from the fact that the model was trained on speech directed to younger children than the ones tested in the experiment.

Taken together, the comparisons with experimental results suggest that the semantic role prediction models capture children's performance relatively well, even though our models are trained on considerably less data than the children are exposed to and the data cover slightly different age ranges in Tagalog and Turkish.

### 3.2 Role predictability in CDUs

We now discuss the results of the semantic role prediction model trained and evaluated on the CDU dataset. Overall, we find that the A role is consistently highly predictable across all positions and languages, whereas the predictability of the P role varies by position and language.

**Fig. 5:**
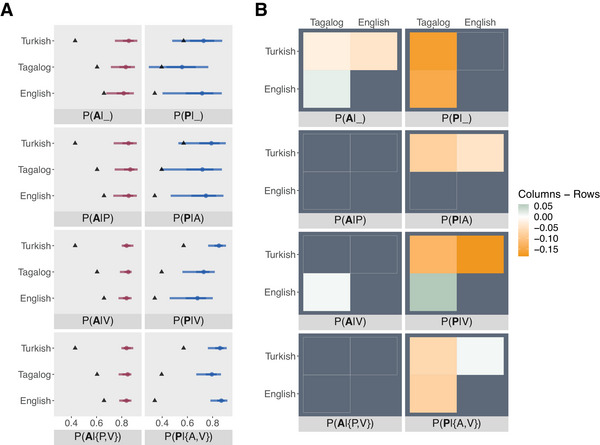
**(A)** Model predictions when fitted on the CDU datasets. Posterior distributions (mean, 66% and 95% intervals) of estimated probability of correct semantic role (“role predictability”) for each language and role in a particular position (e.g., A**P** is the estimated role predictability of P after having seen A). The triangles represent the baseline accuracies according to the proportions of As and Ps in the datasets. For instance, in Turkish, the proportion of A arguments across positions is 43%, resulting in a 43% baseline accuracy for As and a 57% accuracy for Ps, respectively. The estimated role predictability of A is above the baselines already when only A is known, but credibility intervals shrink with more information known, that is, the parser becomes more certain of the A role interpretation with more information at hand. The P role, on its own, is not predicted accurately in Tagalog and in Turkish, with credible intervals strongly overlapping with the baseline. The presence of the verb increases the confidence of predicting P for all languages (P(**P**|V)), and credible intervals shrink even more when *A* is also known (P(**P**|{A,V})). **(B)** Pair‐wise fitted differences of the role predictability between all languages (posterior mean $P\left(\text{correct role}|{\text{language}}_{\mathrm{column}}\right)-P\left(\text{correct role}|{\text{language}}_{\mathrm{row}}\right)$). Differences with support smaller than 95% of the probability mass above 0 are colored gray. The largest effect sizes can be found with the P role. The role predictability the P role in English is lower than the other two languages after either the other argument (panel P(**P**|A)) or the verb (panel PP|V).

Fig. 5A shows the posterior probability of the correct roles (“role predictability”) for each position and language. The first row includes the role predictability of the argument in first position, that is, before the verb and a possible other argument have been seen (facets P(**A**|_) and P(**P**|_)). The second row includes the role predictability after having processed the other argument (facets P(**A**|P) and P(**P**|A)) and the third row after having processed the verb but not the possible other argument (facets P(**A**|V) and P(**P**|V)). The last row shows the role predictability after having encountered both the verb and the other argument in either of the two possible orders (e.g., facet P(**A**|{P,V}) can be either P(**A**|P,V) or P(**A**|V,P)). The triangles represent the baseline accuracies based on a naïve baseline, where the classifier predicts labels according to their proportions in the datasets. For instance, in Turkish, the proportion of A arguments across positions is 43%, resulting in a 43% baseline accuracy for As and a 57% accuracy for Ps, respectively. For English, the proportion of As is 65.9%, and for Tagalog, it is 60.4%. The differences between languages emerge because of the varying degree of argument omission in the respective languages.

The A role is predicted above chance already in the first position when neither the verb nor the P argument are known (facet *P(* **A** *|_)*: posterior mean P(correct A|Tagalog)=0.82,95%CI=[0.71,0.90]; posterior mean P(correct A|English)=0.81,95%CI=[0.68,0.90]; posterior mean P(correct A|Turkish)=0.85,95%CI=[0.75,0.92]). With increasing information, the credibility intervals shrink, particularly after the verb has been seen (*P(* **A** *|V)*: posterior mean P(correct A|Tagalog)=0.85,95%CI=[0.78,0.88]; posterior mean P(correct A|English)=0.84,95%CI=[0.77,0.88]; posterior mean P(correct A|Turkish)=0.84,95%CI=[0.80,0.89]). In fact, the posterior probabilities are virtually the same when only the verb is known or when the verb as well as the P argument are known (facet *P(* **A** *|P,V)*: posterior mean P(correct A|Tagalog)=0.84,95%CI=[0.77,0.88]; posterior mean P(correct A|English)=0.83,95%CI=[0.78,0.88]; posterior mean P(correct A|Turkish)=0.84,95%CI=[0.80,0.89]).

P arguments are predicted with similar accuracies to the baseline when neither the verb nor the A argument are available to the parser in Tagalog and Turkish (facet *P(* **P** *|_)*: posterior mean P(correct P|Tagalog)=0.55,95%CI=[0.29,0.77]; posterior mean P(correct P|Turkish)=0.71,95%CI=[0.48,0.88]). In the same position in English, the 95% CIs do not overlap with the baseline (facet *P(* **P** *|_)*: posterior mean P(correct P|English)=0.70,95%CI=[0.40,0.87]). After the A argument, the role predictability of P increases for all languages, but the 95% CI still overlaps with the baselines for Tagalog and Turkish (facet *P(* **P** *|A)*: posterior mean P(correct P|Tagalog)=0.70,95%CI=[0.38,0.87]; posterior mean P(correct P|English)=0.73,95%CI=[0.47,0.89]; posterior mean P(correct P|Turkish)=0.77,95%CI=[0.53,0.90]). After seeing the verb, the role predictability strongly increases for Turkish and the estimates are more certain (facet *P(* **P** *|V)*: posterior mean P(correct P|Turkish)=0.85,95%CI=[0.76,0.91]). For Tagalog, the mean is lower than for Turkish, but 95% CIs no longer include the baseline (facet *P(* **P** *|V)*: posterior mean P(correct P|Tagalog)=0.72,95%CI=[0.56,0.82]). In English, the posterior probabilities of the P role after the verb remain similar to when only the other argument is known (facet *P(* **P** *|V)*: posterior mean P(correct P|English)=0.67,95%CI=[0.46,0.80]). Finally, once both the verb and the A arguments are known, the P argument is predicted above the baselines with substantial certainty in all languages (facet *P(* **P** *|A,V)*: posterior mean P(correct P|Tagalog)=0.79,95%CI=[0.67,0.87]; posterior mean P(correct P|English)=0.86,95%CI=[0.78,0.92]; posterior mean P(correct P|Turkish)=0.85,95%CI=[0.76,0.91]).

In order to assess differences between languages, we display pair‐wise fitted differences of the role predictability between languages in each position (panel **B** in Fig. 5). Differences with support smaller than 95% of the probability mass above 0 are colored gray. The differences for the A argument are small, with effect sizes ranging between 0.003 and 0.04. The differences for the A argument in the position *P(* **A** *|_)* are highest, with a posterior mean of −0.03 (95% CI = [−0.04, −0.01]) for Tagalog–Turkish, 0.016 (95% CI = [−0.001, 0.03]) for Tagalog–English, and −0.04 (95% CI = [−0.05, −0.03]) for English–Turkish. This indicates that without any further information, the A argument is best predicted in Turkish, followed by English and then Tagalog.

The differences are generally stronger for the P argument. The P argument alone is best predicted in Turkish and least well predicted in Tagalog (facet *P(* **P** *|_)*: posterior mean P(correct P|Tagalog)−P(correct P|Turkish)=−0.16,=[−0.19,−0.14]; P(correct P|Tagalog)−P(correct P|English) = −0.15, 95% CI = [−0.17, −0.12]). After seeing the A argument, the P argument is still best predicted in Turkish (facet *P(* **P** *|A)*: posterior mean P(correct P|Tagalog)−P(correct P|Turkish)=−0.07,95%CI=[−0.13,−0.02]; posterior mean P(correct P|English)−P(correct P|Turkish)=−0.04,95%CI=[−0.07,−0.02]). The strongest pair‐wise differences between languages can be found with the P argument occurring after the verb, where the P argument is by far the most easily predicted in Turkish, followed by Tagalog and the least well in English (facet *P(* **P** *|V)*: posterior mean P(correct P|Tagalog)−P(correct P|Turkish)=−0.13,95%CI=[−0.14,−0.11]; posterior mean P(correct P|English)−P(correct P|Turkish)=−0.17,95%CI=[−0.22,−0.14]; posterior mean P(correct P|Tagalog)−P(correct P|English)=0.05,95%CI=[0.01,0.1]). When both the verb and the A argument are available, the P argument is the least well predicted in Tagalog (facet *P(* **P** *|A,V)*: posterior mean P(correct P|Tagalog)−P(correct P|Turkish)=−0.065,95%CI=[−0.075,−0.056]; P(correct P|Tagalog)−P(correct P|English)=−0.08,95%CI=[−0.09,−0.07]). The difference between English and Turkish is small (facet *P(* **P** *|AV)*: posterior mean P(correct P|English)−P(correct P|Turkish)=0.01,95%CI=[0.002,0.02]).

## 4 Discussion

We examined how children develop an agent preference given naturalistic CDUs and the diversity in role cues across languages with different word orders: Tagalog (verb‐initial), English (verb‐medial), and Turkish (verb‐final).

Our analysis of incremental role predictability showed that the A role is consistently easy to predict across all positions and in all three languages. In particular, estimates from the CDU datasets indicated that A remains highly predictable even in the absence of additional contextual information. This holds true also for Turkish, where the majority of sentences are not A‐initial (see Fig. 1). Taken together, these findings suggest that assigning A roles in child‐directed speech is an easy task which requires little to no contextual support.

Our findings are consistent with the agent preference observed in adult sentence processing and event cognition, where agents are projected into events with minimal contextual input. Strikingly, the agent preference is found in adult processing even after adjusting for any context‐driven lexical expectations (Huber et al., 2024; Sauppe et al., 2023). This agent preference has also been found outside language when subjects exposed to an event depiction for less than 300 ms without any additional context still assign an agent role (Cohn & Paczynski, 2013; Isasi‐Isasmendi et al., 2023). Importantly, this bias emerges early in ontogeny. By 5‐month‐old infants already show a preference for the A role when observing chasing actions (Galazka & Nyström, 2016). The same preference is further found in non‐human great apes (Brocard et al., 2024), suggesting that the privileged role of A might be shared with our closest living ancestors. The agent preference likely emerges from how subjects interact with the world, seeking out, and reporting on, agents as the critical point of departure in event cognition (Bornkessel‐Schlesewsky & Schlesewsky, 2009; DeLancey, 1981; Langacker, 2012; Primus, 2010; Spelke & Kinzler, 2007).

Across our language samples, role predictability shows smaller differences between languages for A arguments than for P arguments. This illustrates that the prediction of A arguments is more similar across languages than the prediction of P arguments. In contrast to the A argument, the P argument cannot be confidently predicted when it appears in the first position, even in Turkish, where P‐initial sentences are common and P frequently occurs before the verb without A. The predictability of P only surpasses the baseline in English. Given the infrequency of P before A word orders in English, this result might be counterintuitive. However, *PAV* word orders occur in very specific constructions, *what‐*questions in our dataset (e.g., *What (P) are you (A) doing (V)?*), increasing their predictability. The predictability of P increases once the verb is available, especially in Turkish and to a lesser extent in Tagalog and English. In English, the use of VP orders (without A) is restricted to imperatives and first and second person omissions (“wanna look at this?” (Lieven et al., 2009, 430157)) and may thus be more constrained than in Tagalog and Turkish, where A omission is more prevalent (see word order distributions in Fig. 1). After seeing both the verb and the A argument, P can be accurately predicted in all languages. The order of P after A and V is consistent with the most frequent word orders in English (AVP) and Tagalog (VAP), which explains why P is most accurately predicted in English in final position. In Turkish, the addition of A does not improve performance and the verb is sufficient for accurately predicting P. Based on naturalistic input, Turkish‐speaking children may struggle to disambiguate P on its own, but the presence of the verb successfully resolves the role ambiguity with respect to P. Our results indicate that Tagalog‐speaking children can also rely on the verb and the voice marker on the verb to disambiguate P and the additional information of A further reduces uncertainty of P. The relatively high predictability of P in Turkish at the end of the sentence may be due to the high frequency of PV utterances in Turkish child‐directed speech, in addition to the accusative case being a particularly reliable cue for the P role (Aksu‐Koç & Slobin, 1985; Özge et al., 2019, 2015). In comparison, in Tagalog, P omission (i.e., VA word orders) is more frequent in child‐directed speech. Especially with the patient voice, the A argument is often expressed as a pronoun, also when it is an established discourse entity, which is different from Turkish, where As are simply dropped (Atlan, 2013; Adricula, 2023). Due to these differences, children acquiring Tagalog may be less exposed to P arguments than those acquiring Turkish, which affects the processing and the acquisition of semantic roles. In English, argument omission is infrequent, apart from “labile” verbs such as “to eat,” which occur with only an A argument. Therefore, the interpretation of P strongly depends on the presence of the verb and the A argument, since its occurrence alone is rare. However, P‐before‐A word orders may occur only in specific constructions, making P more predictable than its baseline based on its frequency in the dataset.

Sentence processing experiments have shown that both adults and children frequently misinterpret initial arguments as As when they are in fact Ps (e. g., Abbot‐Smith et al., 2017; Garcia et al., 2020). Contrary to this, in child‐directed speech, early occurring arguments are not strictly misinterpreted as As. This is shown by the fact that even when only the first argument is known, P arguments are predicted similar to the baseline accuracy (Tagalog and Turkish) or above the baseline accuracy (English), but never below. Thus, although the overall uncertainty is higher with initial P arguments than A arguments, we do not observe a constant misinterpretation of initial P arguments. This discrepancy between naturalistic utterances and experimental stimuli likely arises because of the nature of the experimental stimuli, which include two lexical arguments that lack clear cues for semantic roles. This is confirmed by the fact that when we apply our semantic role prediction model to the experimental stimuli, it also tends to interpret initial arguments as As in English and Tagalog (see Fig. 4). This pattern is not found with the experimental stimuli in Turkish, which shows that the model has learnt the accusative marker (even if on the initial noun) as a cue for the P argument, confirming that the accusative marker is indeed a strong cue for the P argument as has been previously shown in experimental work (Aksu‐Koç & Slobin, 1985; Göksun et al., 2008; Özge et al., 2015, 2019).

The results of the present study have a number of limitations. Our semantic role prediction model is restricted to the sentential level, whereas comprehenders in naturalistic contexts have access to the discourse context. Future studies may also benefit from training the entire model on CDUs rather than pretraining the model on adult conversations. This could be accomplished by selecting a subset of longer sentences—specifically, those containing verbs—from child language corpora, which would require larger datasets, particularly for languages like Turkish and Tagalog. Another important consideration concerns the architecture of the semantic role prediction model. The question arises whether the same results would be observed on the basis of a different model architecture.

## 5 Conclusion

Our study extended the research on the ontogeny of the agent preference by focusing on how much information is needed to accurately predict semantic roles in CDUs. We showed that in these utterances barely any contextual information is needed to accurately predict agents. With regard to the patient, by contrast, children must adapt their parsing strategies early on to the language‐specific demands and cues for this role. Our study further demonstrates the potential of neural networks as tools for investigating item predictability in online sentence processing and for systematically comparing language acquisition without depending on predefined linguistic features.[Fig cogs70147-fig-0006]

## Funding

The study was supported by the NCCR Evolving Language, Swiss National Science Foundation Agreement No. 51NF40_180888.

## Conflict of interest

The authors report no conflict of interest.

## Figures and Tables

**Fig. A1 cogs70147-fig-0006:**
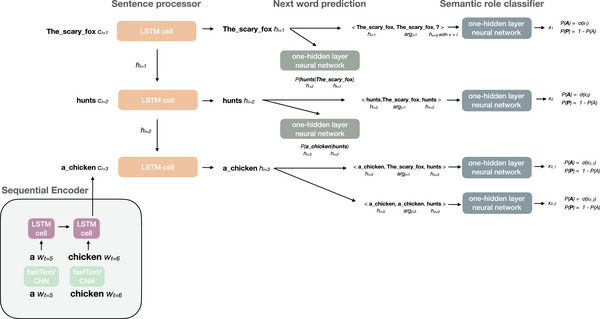
An illustration of the model architecture based on the example sentence ‘The scary dog hunts a chicken’. Words are encoded by the ‘Sequential Encoder’ and then fed to the ‘Sentence processor’, an LSTM, that creates a representation $h_{i}$ from the current representation $c_{i}$ and the preceding representations $c_{0},\text{…}c_{i-1}$. The *semantic role classifier*, a one hidden layer neural network with a softmax function on top, then predicts the role of the already encountered arguments. Additionally, at every time point i, the next representation $x_{i+1}$ is predicted (*next word prediction*).

## Data Availability

The supplementary information, code, and data are available under https://osf.io/e8jk4/?view_only=278b83bd88e040c397a25d25cf7b023f

## Appendix A Experimental Stimuli

**Table A1:** Experimental stimuli from Garcia et al. (2020) and Garcia et al. (2021) crossing *Voice* and *Word Order* conditions.

Voice	Word Order	Verb	Argument 1	Temporal Adverb	Argument 2	Spatial Adverb
Agent Voice	Agent‐initial	H<um>ihila <AV>pull	ang baka SBJ cow	tuwing umaga every morning	ng baboy NSUBJ pig	sa maputik na bukid in muddy LIN field
		‘The cow is pulling a pig every morning in the muddy field.'				
Agent Voice	Patient‐initial	H<um>ihila <AV>pull	ng baboy NSUBJ pig	tuwing umaga every morning	ang baka SBJ cow	sa maputik na bukid in muddy LIN field
		‘The cow is pulling a pig every morning in the muddy field.'				
Patient Voice	Agent‐initial	H<in>ihila <PV>pull	ng baboy NSUBJ pig	tuwing umaga every morning	ang baka SBJ pig	sa maputik na bukid in muddy LIN field
		‘The/A pig is pulling the cow every morning in the muddy field.'				
Patient Voice	Agent‐initial	H<in>ihila <PV>pull	ang baka SBJ cow	tuwing umaga every morning	ng baboy NSUBJ pig	sa maputik na bukid in muddy LIN field
		‘The/A pig is pulling the cow every morning in the muddy field.'				

**Table A2:** Experimental stimuli from Abbot‐Smith et al. (2017) that included the condition of active (agent‐initial) vs. passive (patient‐initial)

Voice	Argument 1	Verb	Argument 2
Active (Agent‐initial)	The girl Agent	is mobbing ACTIVE	the boy Patient
Passive (Patient‐initial)	The girl Patient	is being mobbed PASSIVE	by the boy Agent

**Table A3:** Experimental stimuli from the two experiments reported in Özge et al. (2019). The first experiment included verb‐medial sentences, while the second focused on verb‐final sentences. Both experiments included case marking (nominative (NOM) vs. accusative (ACC)) of the first argument as a condition with nominative case indicating an agent role and accusative case indicating a patient role.

Word Order	Case on initial argument	Argument 1	Temporal Adverb	Verb	Spatial Adverb	Argument 2
Verb‐medial	Nominative (Agent)	Minik tavşan little rabbit.NOM	birazdan soon	yi‐yecek eat‐FUT	şura‐da‐ki there‐LOC‐REL	havuç‐u carrot‐ACC
		‘The little rabbit will soon eat the carrot over there.'				
Verb‐medial	Accusative (Patient)	Minik tavşan‐ı little rabbit‐ACC	birazdan soon	yi‐yecek eat‐FUT	şura‐da‐ki there‐LOC‐REL	tilki fox.NOM
		‘The fox over there will soon eat the little rabbit.'				

Word Order	Case on initial argument	Argument 1	Temporal Adverb	Spatial Adverb	Argument 2	Verb
Verb‐final	Nominative (Agent)	Minik tavşan little rabbit.NOM	birazdan soon	şura‐da‐ki there‐LOC‐REL	havuç‐u carrot‐ACC	yi‐yecek eat‐FUT
		‘The little rabbit will soon eat the carrot over there.'				
Verb‐final	Accusative (Patient)	Minik tavşan‐ı little rabbit‐ACC	birazdan soon	şura‐da‐ki there‐LOC‐REL	tilki fox.NOM	yi‐yecek eat‐FUT
		‘The little rabbit will soon eat the carrot over there.'				

## Appendix B Detailed description of the semantic role prediction model

**Model architecture** Strings in a sentence are first transformed into vector representations $w_{t}$. We tried out two vector encoding methods. First, we used a Convolutional Neural Network (CNN) layer that calculates a representation of a word based on its characters. We used a CNN with six convolutional layers with sigmoid activation functions, each followed by a max pooling layer. We used kernels of sizes 1 to 6 and embedding dimensions for the characters of 100. This resulted in a kernel size of kernel_size × 100. The resulting word embeddings were of size 405. We additionally tried out pretrained fastText embeddings (embedding size = 300), publicly available online (https://fasttext.cc , Grave et al. (2018)). Both of these methods allow for representing words based on their internal structures as opposed to other methods, where words are considered as a whole.

The word representation at time point *t*, $w_{t}$, is first read by the ‘sequential encoder’ implemented as a long short‐term memory (LSTM) unit (see Figure 1). We use one‐layered LSTM units with hidden state dimension size of 100. If the word $w_{t}$, e.g. *the* ($w_{1}$), is the first part of an argument a, e.g., $w_{1}=``{\mathrm{the}}^{\prime \prime }$, $w_{2}=``{\mathrm{scary}}^{\prime \prime }$, $w_{3}=``{\mathrm{dog}}^{\prime \prime }$, the sequential encoder creates a representation of the entire argument by reading in $w_{t}$ until $w_{t+\mathrm{length}(a)-1}$, where length(a) is the number of words in the argument noun phrase (e.g., length(a)=3 in the argument noun phrase $a=``\mathrm{the}\mathrm{scary}{\mathrm{dog}}^{\prime \prime }$). In this way, we ensure that the semantic role is later predicted based on the entire argument, and not just on its head (e.g., *dog*). Words that are not part of arguments are simply passed through the sequential encoder. The output of the sequential encoder at the last time point, $c_{i}$, is then passed to the ‘sentence processor’.

The sentence processor consists of a LSTM unit, again we use a one‐layer LSTM with hidden state dimension size of 100, that calculates a new representation, $x_{i}$, based on the current representation $c_{i}$ and all preceding representations $c_{0},\text{…},c_{i-1}$. This is accomplished by the help of hidden states features in LSTMs. In particular, at each time step, the current word embedding $c_{i}$ and the hidden state from the previous timestep $h_{i-1}$ are passed through a transformation function, resulting in a new hidden state $h_{1}$.

The output representation, $h_{i}$, of the sentence processor is then fed to the semantic role classifier, a one‐hidden layer neural network. The semantic role classifier independently classifies the semantic role(s) of the argument(s) $a_{j}\in X_{i}$ where $X_{i}$ is the set of all arguments that have already been encountered in the sentence until time point i. The input to the classifier is composed of the current representation $h_{i}$ of the sentence processor, a representation of the argument $a_{j}$ and a representation of the verb $h_{v}$ if the verb with time point v has already been encountered at time point i, i.e., v≤i. If the verb has not been encountered yet, i.e v≥i, a zero‐vector is inserted instead. We use a one‐hidden layer neural network with a hidden size of 100 and a sigmoid activation function. We further add the ‘next word predictor’, a language modelling component, again implemented as a one‐hidden neural network classifier with a sigmoid activation function, that is trained to predict the next word, $h_{i+1}$, in the sentence.

**Training** We calculate two types of error that are back‐propagated through the entire model in order to adjust all of the connection weights. For the semantic role classifier, we calculate the cross‐entropy error between the predicted semantic role ${\hat{r}}_{i}$ and the target semantic role $r_{i}$. For the next word prediction classifier, we calculate the *CosineEmbeddingLoss* between the vector of ${\hat{h}}_{i}$ and $h_{i}$. We use a learning rate of 0.001 and batch size of 24. The training unfolds in two stages. We first pretrain the model only on the next word prediction task until the loss does not decrease anymore for three epochs. We then train the model on both the next word prediction and the semantic role classifier. We again train the model until the loss does not decrease anymore for three epochs.

## Appendix C Model definition of the statistical regression model

### C.1 Full model for the main analysis of CDU

We include main effects of position — to be precise, whether the argument occurs after the other argument (*after_arg*) and whether the argument occurs after the verb (*after_verb*) —, the semantic role (*role*), the form of the argument (*nominality*), the verb class of the verb (*verb_class* and interactions between *after_verb* and *after_arg*, *role* and *nominality* as well as *after_verb*, *after_arg* and *role* as main effects. We include a random intercept for the sentence ID (*sent_id*).

We use weakly regularising prior for the population slope: Normal(0,1) and the dispersion parameter ϕ: Exponential(1) or Half‐Normal(0,10). For the by‐sentence variation of the intercept, we use the default priors of brms, a Student‐*t*(3,0,2.5) distribution.

#### C.1.1 *brms* formula

 prior.3.2 <‐ c(prior(normal(0, 1), class = b),

 prior(exponential(1), class = phi))

 prob 1 + after_verb + after_arg + role + nominality + verb_class + after_verb:after_arg + role:nominality + after_verb:after_arg:role + (1|sent_id), family = Beta(), prior = prior.3.2)

#### C.1.2 Model definition

$$\begin{array}{ccc} P(correct) & ∼ & Beta(\mu ,\phi )\\ \text{logit}(\mu ) & = & \alpha +\nu_{\left[\text{sentence},1\right]}+\\ & & (\beta_{1}+\nu_{\left[\text{sentence},2\right]})\times \text{after verb}+\\ & & (\beta_{2}+\nu_{\left[\text{sentence},3\right]})\times \text{after argument}+\\ & & (\beta_{3}+\nu_{\left[\text{sentence},4\right]})\times \text{role}+\\ & & \beta_{4}\times \text{nominality}+\beta_{5}\times \text{verb class}+\\ & & \beta_{6}\times \left(\text{after verb}\times \text{after argument}\times \text{role}\right)+\\ & & \beta_{7}\times \left(\text{after verb}\times \text{after argument}\times \text{language}\right)+\\ & & \beta_{8}\times \left(\text{after verb}\times \text{after argument}\times \text{role}\times \text{language}\right) \end{array}$$

$$\begin{array}{ccc} \beta & ∼ & Normal(0,1)\\ \phi & ∼ & Exponential(1)\\ \left[\begin{array}{c} \nu_{\left[\text{sentence},i=1\right]}\\ \nu_{\left[\text{sentence},i=2\right]}\\ \nu_{\left[\text{sentence},i=3\right]}\\ \nu_{\left[\text{sentence},i=4\right]} \end{array}\right] & ∼ & MVNormal\left(\left[\begin{array}{c} 0\\ 0\\ 0\\ 0 \end{array}\right],\Sigma_{\left[\text{sentence}\right]}\right)\\ \Sigma_{\left[\text{sentence}\right]} & = & S_{\nu }R_{\nu }S_{\nu }\\ R_{\nu } & ∼ & \text{LKJ}(1)\\ S_{\nu } & = & \left(\begin{array}{cccc} \sigma_{\nu_{1}} & 0 & 0 & 0\\ 0 & \sigma_{\nu_{2}} & 0 & 0\\ 0 & 0 & \sigma_{\nu_{3}} & 0\\ 0 & 0 & 0 & \sigma_{\nu_{4}} \end{array}\right)\\ R_{\nu } & = & \left(\begin{array}{cccc} 1 & \rho_{\nu_{1},\nu_{2}} & \rho_{\nu_{1},\nu_{3}} & \rho_{\nu_{1},\nu_{4}}\\ \rho_{\nu_{2},\nu_{1}} & 1 & \rho_{\nu_{2},\nu_{3}} & \rho_{\nu_{2},\nu_{4}}\\ \rho_{\nu_{3},\nu_{1}} & \rho_{\nu_{3},\nu_{2}} & 1 & \rho_{\nu_{3},\nu_{4}}\\ \rho_{\nu_{4},\nu_{1}} & \rho_{\nu_{4},\nu_{2}} & \rho_{\nu_{4},\nu_{3}} & 1 \end{array}\right)\\ \sigma_{\nu } & ∼ & \text{student}\_t(3,0,2.5) \end{array}$$

## Appendix D Model comparison

We assess model performance with leave‐one‐out cross‐validation by means of the expected log pointwise predictive density (*elpd*), approximated with Pareto‐smoothed importance sampling (PSIS) from the posterior log probabilities (Vehtari et al., 2017; McElreath, 2020). We then use model stacking to assess the individual performance of models. Model stacking assigns weights to models to jointly maximise prediction accuracy (Yao et al., 2018) and has become a substitute for other approaches like the AIC and variants thereof (Yao et al., 2018; Höge et al., 2020; Bürkner et al., 2021). Higher weights of a model thus indicate that this model contributes better predictions than models with lower weights.

Model stacking assigned 98% of the weight to the full regression model, 2% to the one without position with regard to the other argument and zero weight to the remaining other two models. The argument's position and language are thus highly relevant for the predictability of roles over the course of a sentence.

**References**

Abbot‐Smith, K., Chang, F., Rowland, C., Ferguson, H., & Pine, J. (2017). Do two and three year old children use an incremental first‐NP‐as‐agent bias to process active transitive and passive sentences?: A permutation analysis. PLOS ONE, 12(10), e0186129. doi: 10.1371/journal.pone.0186129

Bürkner, P.‐C., Gabry, J., & Vehtari, A. (2021). Efficient leave‐one‐out cross‐validation for Bayesian nonfactorized normal and Student‐t models. Computational Statistics, 36(2), 1243–1261. (Publisher: Springer)

Garcia, R., Garrido Rodriguez, G., & Kidd, E. (2021). Developmental effects in the online use of morphosyntactic cues in sentence processing: Evidence from Tagalog. Cognition, 216, 104859. doi: 10.1016/j.cognition.2021.104859

Garcia, R., Roeser, J., & Höhle, B. (2020). Children.s online use of word order and morphosyntactic markers in Tagalog thematic role assignment: an eye‐tracking study. Journal of Child Language, 47(3), 533–555. doi: 10.1017/S0305000919000618

Grave, E., Bojanowski, P., Gupta, P., Joulin, A., & Mikolov, T. (2018). Learning word vectors for 157 languages. In Proceedings of the international conference on language resources and evaluation (lrec 2018).

Höge, M., Guthke, A., & Nowak, W. (2020). Bayesian model weighting: The many faces of model averaging. Water, 12(2). (Number: 309) doi: 10.3390/w12020309

McElreath, R. (2020). Statistical rethinking: A Bayesian course with examples in R and Stan. Chapman and Hall/CRC. Vehtari, A., Gelman, A., & Gabry, J. (2017). Practical Bayesian model evaluation using leave‐one‐out cross‐validation and WAIC. Statistics and computing, 27(5), 1413–1432.

Yao, Y., Vehtari, A., Simpson, D., & Gelman, A. (2018). Using stacking to average bayesian predictive distributions (with discussion). Bayesian Analysis, 13(3), 917–1007. doi: 10.1214/17‐BA1091

Özge, D., Küntay, A., & Snedeker, J. (2019). Why wait for the verb? Turkish speaking children use case markers for incremental language comprehension. Cognition, 183, 152–180. doi: 10.1016/j.cognition.2018.10.026
